# Collinearity Analysis and High-Density Genetic Mapping of the Wheat Powdery Mildew Resistance Gene *Pm40* in PI 672538

**DOI:** 10.1371/journal.pone.0164815

**Published:** 2016-10-18

**Authors:** Shengfu Zhong, Lixia Ma, Syeda Akash Fatima, Jiezhi Yang, Wanquan Chen, Taiguo Liu, Yuting Hu, Qing Li, Jingwei Guo, Min Zhang, Li Lei, Xin Li, Shengwen Tang, Peigao Luo

**Affiliations:** 1 State Key Laboratory of Plant Breeding and Genetics, Sichuan Agricultural University, Chengdu, Sichuan, China; 2 State Key Laboratory for Biology of Plant Diseases and Insect Pests, Institute of Plant Protection, Chinese Academy of Agricultural Sciences (CAAS), Beijing, China; 3 Department of Biology and Chemistry, Chongqing Industry and Trade Polytechnic Institute, Fuling District of Chongqing, China; 4 Department of Agronomy and Plant Genetics, University of Minnesota, Saint Paul, MN, United States of America; GERMANY

## Abstract

The wheat powdery mildew resistance gene *Pm40*, which is located on chromosomal arm 7BS, is effective against nearly all prevalent races of *Blumeria gram*inis f. sp *tritici* (*Bgt*) in China and is carried by the common wheat germplasm PI 672538. A set of the F_1_, F_2_ and F_2:3_ populations from the cross of the resistant PI 672538 with the susceptible line L1034 were used to conduct genetic analysis of powdery mildew resistance and construct a high-density linkage map of the *Pm40* gene. We constructed a high-density linkage genetic map with a total length of 6.18 cM and average spacing between markers of 0.48 cM.*Pm40* is flanked by *Xwmc335* and *BF291338* at genetic distances of 0.58 cM and 0.26 cM, respectively, in deletion bin C-7BS-1-0.27. Comparative genomic analysis based on EST-STS markers established a high level of collinearity of the *Pm40* genomic region with a 1.09-Mbp genomic region on *Brachypodium* chromosome 3, a 1.16-Mbp genomic region on rice chromosome 8, and a 1.62-Mbp genomic region on sorghum chromosome 7. We further anchored the *Pm40* target intervals to the wheat genome sequence. A putative linear index of 85 wheat contigs containing 97 genes on 7BS was constructed. In total, 9 genes could be considered as candidates for the resistances to powdery mildew in the target genomic regions, which encoded proteins that were involved in the plant defense and response to pathogen attack. These results will facilitate the development of new markers for map-based cloning and marker-assisted selection of *Pm40* in wheat breeding programs.

## Introduction

Powdery mildew, which is caused by *Blumeria graminis* f. sp *tritici* (*Bgt*), is a globally destructive disease of common wheat (*Triticum aestivum* L.). This disease often causes large yield and quality losses. Chemical methods are widely used to combat the disease, but identifying new genes in resistant cultivars and deploying those genes in breeding programs would facilitate the development of environmentally safe and effective methods of disease control. New resistance genes must be identified for resistance breeding in wheat. Approximately 79 formally designated powdery mildew resistance alleles have been identified in 50 loci in wheat (*Pm1*-*Pm54*, *Pm18 = Pm1c*, *Pm22 = Pm1e*, *Pm23 = Pm4c*, *Pm31 = Pm21*, *Pm48 = Pm46*) [1–7]. However, many resistance genes often become ineffective due to the enrichment and variation of virulent races, particularly if a single gene is used in large areas and for long periods of time [8]. Therefore, the screening and identification of effective resistance genes and the development of multiple-resistance cultivars are important tasks in wheat breeding.

Alien gene transfer is an extremely efficient approach to increasing the genetic diversity of disease resistance in common wheat. For example, *Pm1b* in line MocZlatka and *Pm1d* in accession TRI 2258, located on chromosome 7A, were transferred from *T*. *monococcum* and *T*. *spelta* to common wheat, respectively [3]. *Pm5a*, located on chromosome 7BL, appears in the variety Hope, which was used extensively as a parent in wheat breeding programs [9]. The dominant allele *Pm4a*, which is located on chromosome 2AL in variety Chancellor [10], and *Pm50*, which is also located on chromosome 2AL, were transferred from *T*. *dicoccum* to common wheat [11]. Several resistance genes have been identified from *T*. *dicoccoides*, including *Pm16*, *Pm26*, *Pm30*, *Pm36*, *Pm41* and *Pm42* [12–17]. In addition, more distantly related species, such as *Secale cereale*, *Haynaldia villosa* and *Thinopyrum intermedium*, are valuable components of the wheat gene pool [18–20].

The wheat line YU25, produced from a wide cross of wheat cultivar CM107 and octoploid Trititrigia TAI7047 (Taiyuan 768/ *Thinopyrum intermedium*//76), exhibits strong resistance against wheat powdery mildew. Genetic analysis demonstrated that the resistance to wheat powdery mildew in YU25 was controlled by the genes *PmE* and *PmYU25*, which are located on chromosomes 7B and 2D, respectively [21]. Finally, we also examined a homogenous wheat line, GRY 19, which only contains *PmE*, which was preliminary mapped on chromosome arm 7BS by five SSR (simple sequence repeat) markers and formally named *Pm40*, the first reported wheat powdery mildew resistance allele transferred from *Th intermedium* [22]. *PmYU25* was originally assigned to 2DL but was mapped on 2BS and named *PmL962* using the SSR and EST-STS markers and Chinese Spring nullisomic-tetrasomic methods [23]. From agronomical traits and application standpoints of view, we developed the following four spring wheat lines: L658 (PI 672537), L693 (PI 672538), L696 (PI 672539), and L699 (PI 672540). These lines exhibited excellent agronomic traits, including yield and resistance to *Fusarium* head blight conferred by *FhbL693a* and *FhbL693b* [24], to stripe rust conferred by *YrL693* [25] and to powdery mildew conferred by *Pm40* [22, 23, 26, 27]. Though the pedigree of PI 672538 includes *Th*. *intermedium*, there is no obvious evidence to demonstrate that *Pm40* was directly originated from *Th*. *intermedium* [22]. For standpoint of the application, the origination of resistance genes is not interesting thing for breeders.

To accelerate the application of *Pm40* in wheat breeding programs, we have explored the underlying molecular mechanism of resistance to powdery mildew as conferred by the host gene *Pm40*, including studying the physiological changes and gene expression profile of *Pm40* during host-pathogen interaction [27]. Although we have constructed genetic linkage maps of *Pm40*, the exact gene location is still unclear, and gene-based markers, such as EST-STS markers, for molecular marker-assisted selection remain elusive. For using *Pm40* in common wheat breeding programs, the construction of a high-density genetic map is essential.

Newly emerged mildew isolates have rendered ineffective many race-specific *Pm* genes, including *Pm2*, *Pm2a*, *Pm5*, *Pm6*, and *Pm8*, that were previously successfully applied in wheat resistance breeding programs in China [28]. *Pm21* still exhibits strong resistance to powdery mildew and is the most commonly employed powdery mildew resistance gene in Chinese breeding programs [19], but new isolates that are highly virulent to *Pm21* have been reported [29,30]. However, *Pm40* confers strong resistance to powdery mildew in the field of both Henna and Sichuan Provinces of China [21,25–26]), and this gene is effective against nearly all isolates collected from the main wheat growing regions of China [23]. Thus, *Pm40* may be widely used in future Chinese breeding programs as an alternative to *Pm21*, necessitating the identification and prioritization of molecular markers closely associated with *Pm40* by marker-assisted selection is an important task.

Comparative genomics analysis is also useful for the development of new molecular markers linked to targeted genes. Comparative genomics analysis has been applied in hexaploid wheat, which has a large genome and numerous repetitive DNA sequences and lacks assembled reference genome sequences [31]. The ESTs can not only be developed into EST-sequence-tagged site (STS) markers and used to construct high-density maps but can also be used in comparative genomics analysis with the available genome sequences of rice (International Rice Genome Sequencing Project 2005), sorghum [32], and *Brachypodium distachyon* (The International *Brachypodium* Initiative 2010). The International Wheat Genome Sequencing Consortium (IWGSC) published a chromosome-based draft of common wheat genomic sequence that makes it possible to search the wheat genome of the regions containing *Pm40* combined with comparative genomics analysis [33]. Via comparative genomics analyses, several disease resistance genes in wheat have been used in map-based cloning, such as *Lr34/Yr18/Pm38* [34] and the stripe rust resistance gene *Yr36* [35].

In this research, to achieve the eventual objective of marker-assisted selection and map-based cloning, we studied the inheritance of *Pm40*, constructed a high-density genetic linkage map of *Pm40*, performed comparative genomics analysis of the regions of *Pm40* in PI 672538, and obtained 9 candidate genes related to powdery mildew resistance.

## Materials and Methods

### Ethics Statement

All the field experiments were permitted by Sichuan Agricultural University (SICAU) and only tested in the experimental plots owned by SICAU. Collecting and inoculating *Bgt* races did not involve endangered or protected species.

### Plant Materials

Two wheat lines, the powdery mildew resistant line PI 672538 [26] that carries the *Pm40* gene without *PmL962* [27] and the susceptible line L1034, were selected from the F_7_ populations of a cross between the susceptible line MY11 and the resistant line YU25. Powdery mildew resistance in YU25 is putatively derived from *Th*. *intermedium* [36,37]. A set of 46 F_1_ plants, 601 F_2_ populations and 579 F_2:3_ lines from a cross of PI 672538/L1034 were used to conduct genetic analysis of the response to powdery mildew and construct a high-density linkage map of the *Pm40* gene.

### Powdery Mildew Evaluations

The prevailing local isolate *Bgt15*, collected from Yaan City, Sichuan province, was used to inoculate the parents and the genetic populations by dusting conidia at a density of 100–140 conidia/mm^2^. The *Bgt15* was avirulent on materials carrying the *Pm40* gene and virulent on MY11 [22]. The wheat seedlings were sown in pots (3 cm diameter) in a growth chamber (Microclima MC1750E, Snijders Scientific, Tilburg, Holland) under controlled conditions with a 14-h light period at 22°C and a 10-h dark period at 18°C for the day/night cycle. Wheat plants were inoculated with *Bgt15* at three-leaf stage seedlings. The same five-week-old seedlings were transplanted on the field to reassess the responses to powdery mildew. Twenty-five hybrid seedlings were planted in a randomized design in 2.5-m rows with 30-cm spacing. The infection types were classified using a rating scale of 0 to 4 [38]. The infection types produced on plants or lines were recorded 3 times over a one-week interval after inoculation.

### DNA Extraction and Bulked Segregant Analysis

Genomic DNA was extracted from seedling leaves using a previously described CTAB protocol [39]. A mixture of equal amounts of bulked DNA from 10 homozygous resistant and 10 homozygous susceptible F_2_ individuals (genotypes based on the reactions of the F_2:3_ lines) was used for bulked segregant analysis (BSA) [40]. The polymorphic markers between the resistant and susceptible parents and the bulked DNA were chosen to genotype the F_2:3_ lines to construct the linkage map of *Pm40*.

### Polymerase Chain Reaction (PCR)

For the initial polymorphic marker survey, *gwm* [41] and *wmc* [42] SSR markers located on the wheat chromosome 7B according to a previously constructed consensus map [43] were selected and used in BSA to screen for markers linked to the resistance gene. PCR (25-μl volume) was performed in a PTC-200 thermocycler (MJ Research, Watertown, MA, USA). SSR analysis was performed following a previously described procedure [41] with minor modifications. Each PCR mixture contained each SSR primer at a concentration of 200 nmol/L, 0.2 mmol/L dNTPs, 1.5 mmol/L MgCl_2_, 1 unit of Taq polymerase, and 60 ng of template DNA. PCR was performed following a previously described program [23]. Then, 4 μL of each PCR product was mixed with 2 μL of loading buffer and loaded onto a 6% non-denaturing polyacrylamide gel for separation and visualization by silver staining [44].

### Development of EST-STS Markers

To increase the marker density of the map, we chose other published SSR markers located on chromosome 7BS that co-segregated with the resistance locus in BSA, but other SSR markers were tested that were not linked with *Pm40*. Based on the published locations of the six linked SSR markers on wheat chromosome 7BS, the *Pm40* genetic map region was located on the wheat C-7BS-1-0.27 bin map [45]. A total of 67 EST-STS markers were developed based on ESTs mapped on chromosome deletion 7BS bin 1–0.27 using the software primer3 [46]. These markers were employed to screen polymorphisms between the resistant and susceptible bulked DNA to construct a high-density genetic map and identify orthologous genomic regions.

### High-Density Genetic Linkage Map Construction

We performed Chi-squared (χ2) tests for goodness-of-fit of the segregation data with theoretically expected segregation ratios of 1:2:1 or 3:1 using Sigmaplot 2001 software (SPSS Inc., Chicago, IL, USA). Recombination fractions were converted to map distances (cM) using the Kosambi mapping function [47]. Loci exhibiting no significant deviations (*P* > 0.05) were used in the linkage analysis. Linked molecular markers and the *Pm40* locus were determined using JoinMap 4.0 with a LOD threshold of 3.0.

### Comparative Genomics Analysis

The polymorphic EST-STS markers between PI 672538 and L1034 as well as the resistant and susceptible bulked DNA were used to identify orthologous gene pairs. The markers linked with *Pm40* and corresponding EST sequences were analyzed by BLASTn at a 10^−5^ threshold probability against the genome sequence databases of *Brachypodium distachyon*, *Oryza sativa japonica* and *Sorghum bicolor*. After putative highly conserved gene pairs were obtained, we continued to perform tBLASTx to conduct comparative genomic analysis of the genomic regions on both sides of orthologous gene pairs among *Brachypodium distachyon*, *Oryza sativa japonica* and *Sorghum bicolor*. Genomic regions with s high level of collinearity were identified as orthologous genomic regions containing the *Pm40* locus and polymorphic EST-STS markers. Furthermore, the sequences of those genes in three genomic regions were used as queries for BLASTn at a 10^−5^ probability threshold and minimum of 100 bp match length against a chromosome-based draft of the wheat genomic sequence “Assembly_MIPSv2REF_Bgenome_cleaned_rep-masked” from IWGSC (http://www.wheatgenome.org/). Then, the contigs with the best hit were employed for searching the remaining non-homologous genes via IWGSC genomic annotation. Functional annotation of genes was performed using the software Blast2GO 3.30. Genomic locations were determined *in silico* using the software Circos 0.6.4.

## Results

### Inheritance of Resistance to Powdery Mildew in PI 672538

**Fig 1:**
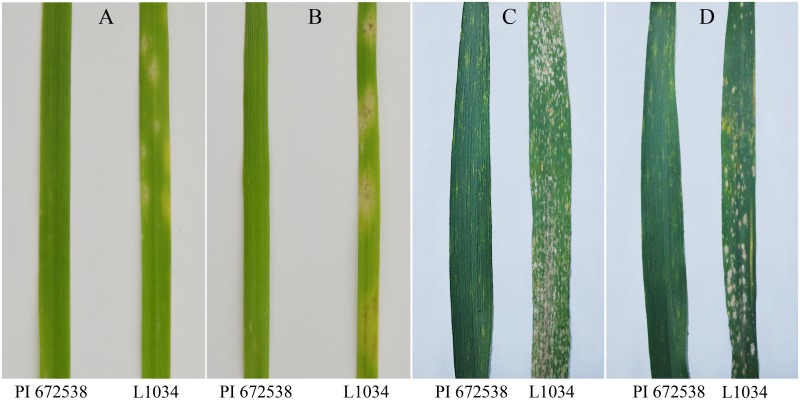
Powdery Mildew Responses on Infected Leaves of Parental Lines. (A) and (B), Infected leaves of PI 672538/L1034 in the growth chamber 14 days (A) and 21 days (B) after inoculation with *Bgt15*. (C) and (D), Infected leaves of PI 672538/L1034 in the field 21 days after inoculation with *Bgt15* in the years 2013 (C) and 2014 (D).

The results of resistance identification confirmed that PI 672538 was resistant and L1034 was susceptible to powdery mildew at both the seedling stage and in adult plants (Fig 1). In total, 46 F_1_ plants, 601 F_2_ populations and 579 F_2:3_ lines from PI 672538/L1034 crosses were inoculated with *Bgt15*. The F_1_ plants were resistant, with responses similar to that of PI 672538. Thus, the resistance was dominant. The F_2_ population segregated as 450 resistant and 151 susceptible. The F_2:3_ lines segregated as 144 resistant, 145 susceptible, and 290 segregating in response to *Bgt15* (Table 1). These data fit the single Mendelian locus ratio (χ2 3:1 = 0.005, *P*>0.05; χ2 1:2:1 = 0.005, *P*>0.05), indicating that PI 672538 powdery mildew resistance is controlled by a single dominant gene.

**Table 1:** Phenotypes of F_1_, F_2_, and F_2:3_ Populations Obtained from PI 672538/L1034 with the *Bgt15* Isolate.

Generation	Observed numbers of F_1_, F_2_ and F_2:3_ lines			Expected ratio	χ^2^^a^	*P*
	Resistant	Segregating	Susceptible			
F_1_	46					
F_2_	450		151	3:1	0.005	0.943
F_2:3_	144	290	145	1:2:1	0.005	0.997

^a^ Value for significance at *P* = 0.05 is 3.84

### Identification of Microsatellite Markers Linked with *Pm40*

The *Pm40* gene was previously mapped to wheat chromosome 7BS. Thus, 87 published SSR markers mapped to wheat chromosome 7BS were chosen to map the *Pm40* gene. A total of 9 (10.3%) of 87 microsatellite markers were polymorphic between PI 672538 and L1034. Of these, 6 markers, *Xwmc364*, *Xwmc335*, *Xwmc476*, *Xgwm297*, *Xwmc662* and *Xgwm*43, were linked with *Pm40* after genotyping the resistant and susceptible F_2_ bulked DNA.

### Identification of EST Markers and Construction of a Genetic *Pm40* Linkage Map

**Fig 2:**
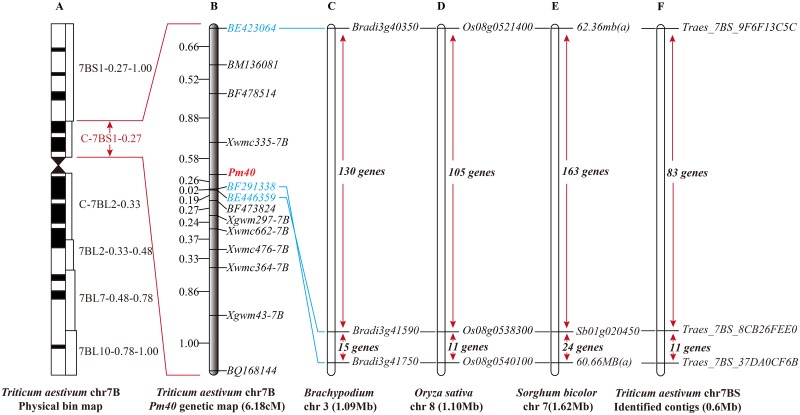
The Genetic and Comparative Genomic Linkage Maps of the Wheat Powdery Mildew Resistance Gene *Pm40*. (A) Physical map of wheat chromosome 7B. (B) Genetic linkage map of *Pm40*. (C) The orthologous genes on *Brachypodium distachyon* chromosome 3. (D) The orthologous genes on *Oryza sativa japonica* chromosome 8. (E) The orthologous genes on *Sorghum bicolor* Chromosome 7. (F) The wheat contigs and genes anchored between the flanking markers on chromosome 7BS. The red line indicates that the *Pm40* genetic map region was included in the wheat C-7BS-1-0.27 bin map. The blue line indicates that the genomic regions of the 1.09-Mb *Brachypodium distachyon* chromosome 3, 1.10-Mb *Oryza sativa japonica* chromosome 8, and 1.62-Mb *Sorghum bicolor* chromosome 7 were homologous with the wheat genetic map region between loci *BE423064* and *BE446359/ BF291338*.

Of 67 EST-STS markers developed from sequences mapped on the chromosome 7B deletion, 7 EST markers were polymorphic between the PI 672538 and L1034 as well as the resistant and susceptible bulked DNA. The sequences of the EST-STS markers linked with *Pm40* are presented in Table 2. The linked EST-STS and previous SSR markers were used to genotype the F_2:3_ populations. The relationship between the *Pm40* gene and the marker genotypes is presented in S1 Table Each marker locus segregated in 1:2:1 or 3:1 ratios. A linkage map spanning chromosome arm 7BS was constructed (Fig 2B). In total, 13 polymorphic markers and *Pm40* were located on the genetic map. The map spans 6.18 cM with an average distance of 0.44 cM between markers. *Pm4*0 is narrowly flanked by the markers *Xwmc335* and *BF291338* with distances of 0.58 and 0.26 cM located in deletion bin C-7BS-1-0.27 (Fig 2A).

**Table 2:** Sequences of the EST-STS Markers Closely Linked with *Pm40*.

Marker name	Forward primer	Reverse primer	Type
*BE423064*	CTGAGTCCAGTGTCCACGAC	CCATTGCAGGCAAACAGTCC	Dominant
*BQ168144*	CCTGCTCGACTCCATGAACA	TCCCTTAGGGTGGTTCAGCT	Codominant
*BE446359*	AGACAAGAACACCCGTGCAA	TGGCAGATTCAGTTGATCGGA	Dominant
*BF473824*	GGAACAGTTATCCGGTGGGG	AACCCAGCAACTTTGGCAAC	Codominant
*BF291338*	GCGCTCTCGGCTCTAGATTT	GAACAATCTTCGCTGCACCC	Dominant
*BF478514*	AAAGAAGGAGCTCACACGGG	CTGGCCGTTCTTCTCCTCTG	Codominant
*BM136081*	GACCATGATCAGGTGCTGCA	TCATTCAGCAACAGCGCATG	Dominant

### Comparative Genomic Analysis and Identification of Candidate Genes

The sequences of 7 EST-STS polymorphic markers flanking *Pm40* were used as queries to search for orthologs in rice, sorghum and *Brachypodium* genomic sequences. The 3 markers *BE423064*, *BE446359*, and *BF291338* are homologous to *Bradi3g40350*, *Bradi3g41590*, *Bradi3g41750* of *Brachypodium*; *Os08g0521400*, *Os08g0538300*, *Os08g0540100* of rice; and *Sb07g025790* of sorghum (Table 3). Based on these 3 orthologous gene pairs, the orthologous genomic regions containing *Pm40* and 3 markers in *Brachypodium*, rice, and sorghum were identified (S2 Table). The relationships between the 3 markers and their orthologous regions are presented in Fig 2B–2E. A high level of genomic collinearity was observed between rice sorghum and *Brachypodium*. Comparative genomic analysis established the collinearity of the *Pm40* genomic region with a 1.09-Mbp genomic region harboring 148 genes on chromosome 3 in *Brachypodium*, a 1.16-Mbp genomic region harboring 119 genes on chromosome 8 in rice,the orthologous gene order was highly conserved among these species (S1 Fig). As such, all of these genes were employed to search and sort the orthologs in wheat chromosome 7BS. Among this 2.92cM region of wheat, a total of 87 loci (76 IWGSC predicted genes and 11 unpredicted) distributed among 85 contigs were identified between markers *BE423064* and *BE446359* (S3 Table). Then, these 0.6-Mbp contigs putatively carrying *Pm40* were employed to identify the genes that were not orthologous with the other 3 species. An additional 10 genes were identified in these contigs.

**Table 3:** Blast Search Results for 3 EST Sequences against Orthologous Regions of the *Brachypodium distachyon*, *Oryza sativa japonica* and *Sorghum bicolor* Genomes.

Species	EST	E-value	Identity(%)	Chromosome	Hit start	Hit end	Gene
*Brachypodium distachyon*	*BE423064*	8.19E^-101^	85.30	3	42517322	42521947	*Bradi3g40350*
	*BE446359*	4.53E^-53^	89.70	3	43500252	43503473	*Bradi3g41590*
	*BF291338*	7.37E-^68^	91.30	3	43599517	43602898	*Bradi3g41750*
*Oryza sativa japonica*	*BE423064*	3.91E^-63^	77.10	8	26025102	26030450	*Os08g0521400*
	*BE446359*	1.56E^-42^	87.70	8	26997132	27001497	*Os08g0538300*
	*BF291338*	7.76E-^64^	89.70	8	27122943	27126791	*Os08g0540100*
*Sorghum bicolor*	*BE423064*	4.00E^-48^	79.10	7	62355820	62356236	no annotation
	*BE446359*	5.20E^-40^	86.30	7	60924644	60929776	*Sb07g025790*
	*BF291338*	1.10E^-59^	88.00	7	60662188	60661860	no annotation

In total, 97 genes were assigned to this region (Fig 2F). The putative functions of these genes are shown in S4 Table. Among them, 82 genes were annotated from the NCBI database through BLASTx search at a 10^−5^ probability threshold. Sixteen out of 82 genes were referred to as ‘‘hypothetical protein” or ‘‘predicted protein”. Nine genes showed the functional annotations that were involved in the disease resistant mechanisms (Table 4). Three out of 9 genes domains include disease resistance proteins RGA1 and RPM1 respectively, which were both annotated as belonging to the Nucleotide Binding Site (NBS)-Leucine Rich Repeats (LRR) class. The remain 6 genes were involved in mechanisms of plant defense and response to environmental stresses such as wounding and pathogen attack, including one of hypothetical proteins related in defense response to fungus (GO: 0050832). These 9 genes could be considered as candidates for the resistances to powdery mildew in target genomic regions.

**Table 4:** Wheat genes involvement in the disease resistant identified at the *Pm40* target intervals.

Wheat Contig	Wheat gene/hit start-end	Annotation	Brachypodium	Rice	Sorghum
ta_iwgsc_7bs_v1_3135063	3045–3786	Disease resistance RGA1	-	-	Sb07g025890
ta_iwgsc_7bs_v1_3162204	5557–6183	Disease resistance RGA1	-	-	Sb07g025850
ta_iwgsc_7bs_v1_3133660	1803–2659	Disease resistance RPM1	-	Os08g0539400	-
ta_iwgsc_7bs_v1_3035032	4425–4616	probable glucan endo-1,3-beta-glucosidase A6	Bradi3g40907	-	Sb07g026540
ta_iwgsc_7bs_v1_3150881	Traes_7BS_17B99F895	Calcium-transporting ATPase plasma membrane-type	Bradi3g40640	-	Sb07g026810
ta_iwgsc_7bs_v1_3133559	Traes_7BS_FD25753C1	Pumilio 5	Bradi3g40510/Bradi3g40520	**-**	**-**
ta_iwgsc_7bs_v1_3165877	Traes_7BS_FF9F03B12	Peroxidase 55	Bradi3g41340	Os08g0532600/Os08g0532700	-
ta_iwgsc_7bs_v1_3166795	Traes_7BS_984D5C4A3	L-ascorbate peroxidase	**-**	Os08g0522400/Os08g0522500	**-**
ta_iwgsc_7bs_v1_3113242	Traes_7BS_F209296D4	hypothetical protein F775_32194	Bradi3g41350	Os08g0532800	-

## Discussion

### The Contribution of the EST-STS Markers to the Construction of the High-Density Genetic Map

To get the candidate resistance genes, constructing a high-density genetic map is essential. ESTs provide abundant information for gene expression profiling. The large number of localized ESTs allows EST-STS markers to be applied in the construction of a high-density genetic map [46]. In contrast to SSR markers, EST-STS markers reflect functional differences in genes and are useful for conducting comparative genomic analyses [48]. *Pm40* was previously located by only five SSR markers encompassing a genetic distance of 10.9 cM using an F_2_ population of 213 individuals [22]. In the present study, we confirmed that four of the five previously identified SSR markers, *Xwmc335*, *Xgwm297*, *Xwmc364* and *Xwmc476*, were also linked with *Pm40* in the mapping populations derived from the PI 672538/L1034 cross. Additionally, two additional linked SSRs markers and seven EST-STS markers were also located with *Pm40* in this research using an F_2_ population of 579 individuals (Fig 2). The average genetic distances between these markers and *Pm40* ranged from 2.18cM to 0.48cM. Furthermore, the newly developed EST-STS markers *BE446359*, *BF473824* and *BF291338* are closer to *Pm40* than the previous flanking marker, *Xgwm297*. Because EST-STS markers are typically located in conserved regions of expressed genes, the most closely linked marker *BF473824*, is more suitable for use in molecular marker-assisted breeding than SSR markers. The distance between the marker *Xwmc335* and *Pm40* increased from 0.2 cM to 0.58 cM [22] (Fig 2); the discrepancies in reported distances between a single marker and *Pm40* may result from differences in the population sizes and genetic backgrounds of the materials studied. The population in the present study included 579 F_2_ individuals, larger than the size of 213 used in previous studies; thus, a high-solution map was constructed to identify candidate resistance genes.

### Identified the Candidate Genes of Powdery Mildew with the Methods of Comparative Analysis

Comparative genomics is a powerful method to study the species without reference assembled genome sequences. Genomic resources for wheat improvement have lagged behind other major crops, such as maize and rice. Draft genome sequences of the wheat A-genome progenitor *Triticum urartu* and the wheat D-genome progenitor *Aegilops tauschii and* a chromosome-based draft sequence of hexaploid bread wheat *(Triticum aestivum)* genome have been reported [33,49,50]. However, sequence assembly and annotations of wheat are not complete. Thus, the EST-STS markers developed from wheat provide an excellent tool for comparative genomics analyses and homologous cloning. In the present work, three EST-STS markers, *BE423064*, *BE446359*, and *BF291338*, were used to identify the orthologous genomic regions containing *Pm40* in *Brachypodium*, rice, and sorghum (Fig 2). As the first sequenced gramineous crop, rice has been successfully subjected to comparative genomics analyses. For example, the stripe rust resistance gene *Yr36* was cloned by analyzing collinear regions in rice chromosome 2 [35]. However, gene rearrangement between the orthologous regions of wheat and rice was observed in the process of cloning the resistance genes *Lr10*, *Lr21* and *Pm3b* [51–53]. In the *Pm40* genomic regions, the synteny levels of the orthologs between rice and *Brachypodium*, rice and sorghum, *Brachypodium* and sorghum are 75.6%/60.8% (90 of 119/148), 79.8%/50.5% (95 of 119/188) and 83.1%/65.4% (123 of 148/188), respectively (Table 3). The collinearity of *Brachypodium* between rice and sorghum is higher than that in the other pairs. Moreover, previous research has indicated higher collinearity between *Brachypodium* and wheat compared with wheat and rice and sorghum based on comparative genomics analysis of disease resistance gene regions [31,54–56]. Thus, the collinear regions of *Pm40* in *Brachypodium* are likely more favorable as a reference to determine the gene order in wheat. By comparing our results of the *Pm40* target intervals to the 7BS Genome Zipper described by IWGSC [33], we found that the order of the wheat genes was similar to that of the anchored *Brachypodium* genes. Unfortunately, we couldn’t anchor the marker *BE423064* to the contig data from the IWGSC Genome Zipper, although a region of 84.95cM to 85.46cM on 7BS was similar to our final results for the collinear region. Therefore, it is difficult to directly use the data from the wheat Genome Zipper in our research, like a previous report regarding the positional isolation of powdery mildew QTLs in barley [57]. Successful fine mapping of quantitative trait loci by using the synteny-based or zipper-based markers combined with primarily genome zipper and population sequencing analysis has been reported [58,59]. Based on previous research, 97 gene were identified according to our synteny analysis with EST-STS markers. By comparison, only about 70 genes were anchored in this region by the IWGSC Genome Zipper analysis. Therefore, according to these EST-STS markers, we obtained a high-solution genomic data to provide more useful information for identifying the candidates of the powdery mildew gene *Pm40*.

Inspection of all predicted proteins located in the target regions within *Pm40* genes, we found 9 candidate genes encoded proteins that were involved in plant resistance (Table 4). Among them, 3 genes disease were identified as belonging to NBS-LRR class, which represents one of the major classes of resistance genes. Like most NBS-LRR resistance proteins, RGA1 and RPM1 guard the plant against pathogens via a direct or indirect protein–protein interaction [60,61]. Two candidates generally were described that defend from pathogen attacks via adjusting the oxidation-reduction reaction, including the genes encoding “glucan Peroxidase 55” and “L-ascorbate peroxidase proteins” [62,63]. Another two genes were annotated as “Calcium-transporting ATPase plasma membrane-type protein” and “probable glucan endo-1,3-beta-glucosidase A6”, which may play an important role in the signaling networks of the pathogen effectors [64,65]. One additional candidate gene encode an equence-specific RNA-binding protein “Pumilio 5” that regulates translation and mRNA stability by binding the 3'-UTR of target mRNAs [66]. That triggers a unique defense system which affects pathogens replication. [67]. The last one is referred to as an uncharacterized protein “hypothetical protein F775_32194”, which identified from *Aegilops tauschii*. It seems that may be involved in defense response to fungus (GO: 0050832) [49]. Apart from these, 30 out of 97 (31%) genes did not hit characterised proteins with predicted functions (S4 Table). These uncharacterised genes also will be concerned in the future work.

### Polymerization and Application of Powdery Mildew Resistance Genes, including *Pm40* Located on Chromosome 7B

The first powdery mildew resistance gene mapped on chromosome 7BS, *Pm40*, is dominant [22], whereas the second powdery mildew resistance gene mapped on 7BS, *Pm47*, is recessive [68]. In addition, *Pm40* was putatively derived from *Thinopyrum intermedium*, whereas *Pm47* was derived from wheat. Molecular linkage marker analysis revealed that the SSR markers linked to *Pm40*, which was physically mapped to bin C-7BS-1-0.27 near the centromere, are different from *Pm47*, which was mapped to bin 7BS-1-0.27–1.0. Based on pedigree, inheritance, molecular marker experiments, and genetic location, these data reveal large genetic differences between *Pm40* and *Pm47*. Other resistant genes, including *Pm5a-Pm5e* [9,53,69], *mlxbd* [70], *PmH* [71], *mljy*, and *mlsy* [72], were also mapped to 7BL. This information indicates that we can develop stable, durable resistance against powdery mildew lines or cultivars by pyramiding the different *Pm* genes via chromosome recombination. Moreover, closely linked markers may accelerate the process of generating recombinant plants. The molecular markers closely linked with the *Pm* gene on 7B, such as EST-STS markers *BF291338* closed linked with *Pm40 and BE606897* closed linked with *Pm47*, may allow a recombinant chromosome with multiple resistance genes to be constructed more rapidly and efficiently [22,68].

### Potential Role of PI 672538 in Marker-Assisted Selection for the Wheat Breeding Program

The resistant line PI 672538 used in the present study was selected from the progeny of MY11 and YU25 [26]. YU25 was derived from the cross between common wheat and *Thinopyrum intermedium* [21,36]. However, genomic *in situ* hybridization demonstrated that there is no alien chromosome fragment in PI 672538 [73]. In addition, in the present research, the responses of PI 672538/L1034 and crosses of the F_1_, F_2_, and F_2:3_ populations to powdery mildew in different growth phases revealed PI 672538 resistance at both the seedling stage and in adult plants (Fig 1). PI 672538 was also simultaneously tested by artificial inoculation and natural inoculation in the field (Fig 1C and 1D). PI 672538 was not only resistant to the race *Bgt15* but also exhibited resistant to various complex localized races. Extensive resistance identification and agronomic characterization of PI 672538 and L1034 have revealed that the effective resistance of PI 672538 to powdery mildew and stripe rust afforded is conferred by the wheat stripe rust resistance gene *YrL693* [36,71] and *Fusarium* head blight resistance [26], respectively. Moreover, PI 672538 also exhibits favorable agronomic and morphological traits [26]. In summary, PI 672538 is an ideal material for resistance breeding. Therefore, the identification of EST-STS markers *BF291338* closely linked to *Pm40* will be beneficial for marker-assisted selection in the wheat breeding program. Comparative genomics analysis of the *Pm40* region may aid further studies aimed at map-based cloning of resistance genes.

## Supporting Information

S1 Fig Comparative genomic mapping of ESTs in the *Pm40* chromosomal region (*BE423064*-*BE446359*). The gene order in all species is clockwise.
(TIF)

S1 Table Marker and powdery mildew response genotypes for the PI 672538/1034 F_2_ populations. (XLSX)

S2 Table The orthologs of the *Pm40* orthologous genomic regions in *Brachypodium*, rice and sorghum. (XLSX)

S3 Table Identified wheat contigs and genes between flanking markers of *Pm40*. Wheat gene order is by determined by orthologs in *Brachypodium*, rice and sorghum.
(XLSX)

S4 Table The functional annotation of all of the genes in the wheat target contigs. (XLSX)
