# Supplementary figures and images for: Collinearity Analysis and High-Density Genetic Mapping of the Wheat Powdery Mildew Resistance Gene Pm40 in PI 672538
Source: PLoS One. 2016 Oct 18;11(10):e0164815. doi: 10.1371/journal.pone.0164815 (PMC5068701; doi:10.1371/journal.pone.0164815)

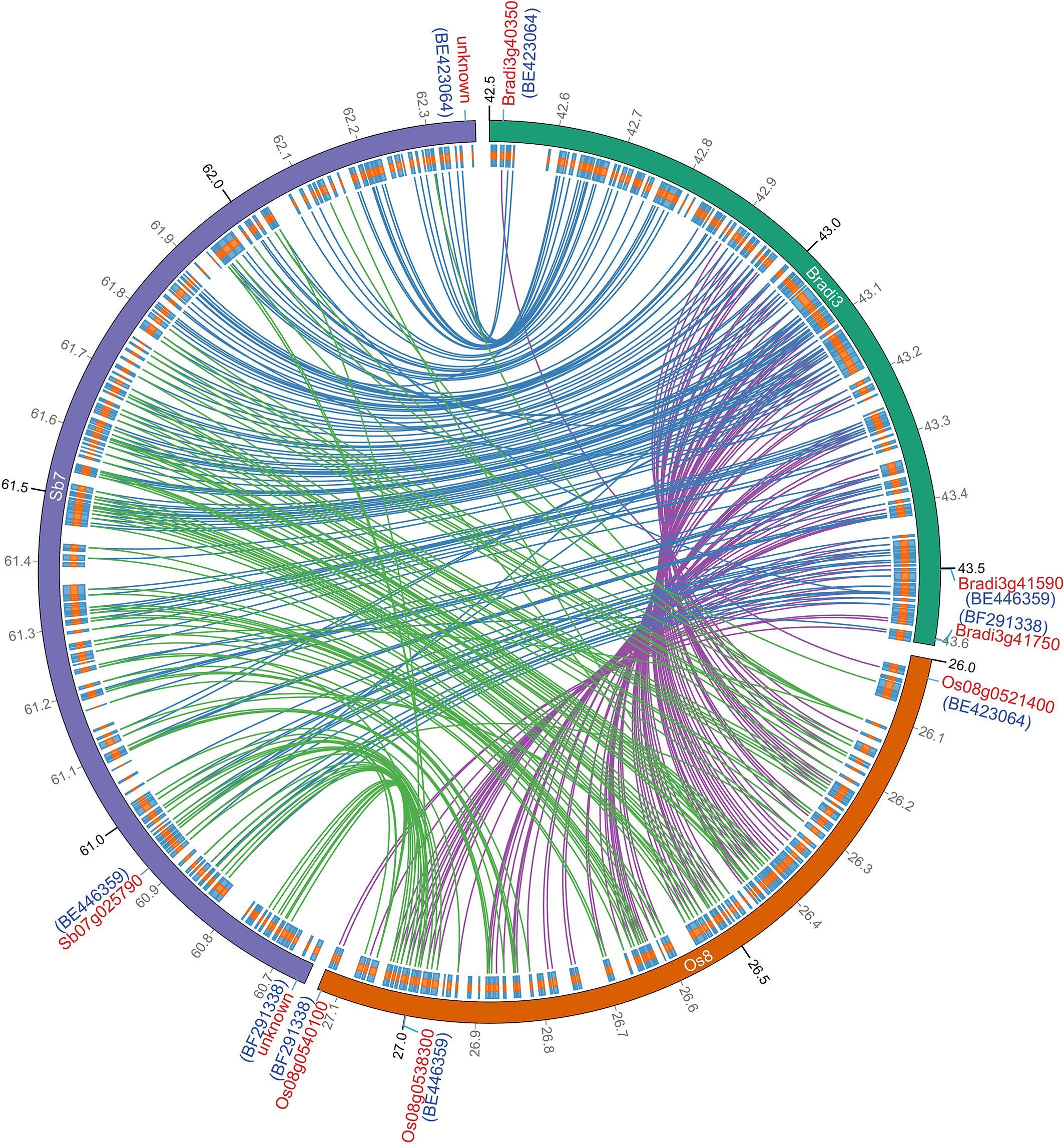

Supplement: S1 Fig — The gene order in all species is clockwise. (TIF) [file pone.0164815.s001.tif]
